# The Association of Upper Airway Anatomy and Hypoglossal Nerve Stimulation Response in OSA

**DOI:** 10.1002/lary.70493

**Published:** 2026-03-19

**Authors:** Chau Phung, Tice Harkins, Linda C. Magaña, Yash Dixit, Eric Thuler, Muskaan Aggarwal, Andrew Wiemken, Everett Seay, Brendan T. Keenan, Bruno Saconi, Richard J. Schwab, Raj C. Dedhia

**Affiliations:** ^1^ Department of Otorhinolaryngology—Head & Neck Surgery University of Pennsylvania Philadelphia Pennsylvania USA; ^2^ Division of Sleep Medicine, Department of Medicine University of Pennsylvania Philadelphia Pennsylvania USA

**Keywords:** hypoglossal nerve stimulation, obstructive sleep apnea, sleep endoscopy, upper airway anatomy

## Abstract

**Objectives:**

Hypoglossal nerve stimulation (HGNS) is a promising surgical treatment of obstructive sleep apnea (OSA) for patients intolerant to positive airway pressure (PAP). Nonetheless, more than one‐third of patients have suboptimal outcomes, demonstrating the need for improved understanding of factors associated with treatment effectiveness. This study examined the relationship between anatomy measures from computed tomography (CT) and HGNS outcomes.

**Methods:**

A prospective cohort study of consecutively enrolled adults with moderate or severe OSA (AHI > 15 events/h) who underwent HGNS implantation between February 2020 and June 2024. Preoperative CT scans were obtained following a standardized protocol. Anatomic traits, including the size of the tongue and the surrounding craniofacial structures, were quantified. HGNS response was defined by the Sher criteria (≥ 50% reduction in apnea–hypopnea index [AHI] and postoperative AHI < 20 events/h).

**Results:**

A total of 65 patients were included: 31 HGNS responders and 34 nonresponders. On average, patients were 63.3 ± 11.7 years old, overweight (BMI of 29.5 ± 4.0 kg/m^2^), about half were male (50.8%) and most were White (89.2%); the average AHI was 28.6 ± 13.3 events/h. No statistically significant associations with HGNS response were observed for relative tongue volume (tongue volume/total oral cavity volume; OR: 0.82 [0.48–1.40]; *p* = 0.46), absolute tongue volume (OR: 1.27 [0.62–2.61]; *p* = 0.52), or total oral cavity volume (OR: 1.81 [0.82, 3.99]; *p* = 0.14). Exploratory analysis showed hyoid position and transverse maxillary dimensions were associated with response status.

**Conclusion:**

Our study did not find a significant association between relative tongue volume and HGNS response. Further investigations in larger samples may elucidate links between HGNS response and upper airway anatomy.

**Level of Evidence:**

4.

## Graphical abstract

CT‐derived measurements of tongue and craniofacial anatomy were evaluated in adults undergoing hypoglossal nerve stimulation for obstructive sleep apnea. Relative tongue volume was not significantly associated with treatment response. Exploratory analyses suggest that other anatomic features such as hyoid positions or transverse maxillary dimensions may contribute to heterogeneous outcomes and warrant further study.

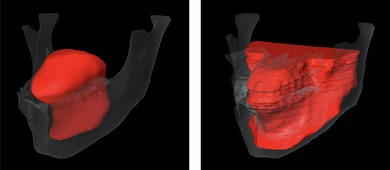

## 1 Introduction

Obstructive sleep apnea (OSA) is a sleep‐related breathing disorder characterized by upper airway obstruction, intermittent hypoxemia, and fragmented sleep [1]. Untreated OSA can lead to long‐term morbidity, including neurocognitive decline and cardiovascular disease, as well as increased mortality [2, 3]. Positive airway pressure (PAP) remains the first‐line therapy for OSA [4]. Nevertheless, more than half of patients do not adhere to PAP therapy, resulting in a need for therapeutic alternatives such as hypoglossal nerve stimulation (HGNS) [5, 6]. In select patients with moderate or severe OSA, HGNS therapy has demonstrated a reduction in apnea–hypopnea index (AHI) and better sleep quality with long‐term use [7]. Despite current selection criteria, including traits from preoperative drug‐induced sleep endoscopy (DISE), one‐third of patients who have HGNS implanted demonstrate a suboptimal clinical response, highlighting the need for a better understanding of factors associated with HGNS response [8].

Previous studies have identified several clinical and physiologic characteristics that associate with outcomes following HGNS therapy. Patients with less severe OSA, typically defined as an AHI < 50 events/h and lower body‐mass index (BMI < 32 kg/m^2^), tend to respond more favorably to HGNS [9, 10, 11]. In addition, the absence of complete concentric collapse of the velopharynx during DISE is associated with better outcomes [12]. PAP levels, assessed during either natural sleep or DISE, may also serve as an indicator of likely success [13, 14]. Responders, as defined by the Sher criteria (≥ 50% reduction in AHI and a postoperative AHI of < 20 events/h), tend to require lower PAP levels (< 8 cmH_2_O) compared to nonresponders [13, 14]. While these clinical characteristics are associated with therapy outcomes, they do not fully account for variability in HGNS response, suggesting that additional factors contribute to HGNS efficacy [15].

In addition to clinical, demographic, and sleep study characteristics, craniofacial and soft tissue anatomy has emerged as crucial, yet underexplored, markers of HGNS response. Using awake computed tomography (CT), Schwab et al. demonstrated that responders have smaller soft palate volume, greater tongue displacement, and reduced hyoid‐to‐mandible distance [16]. Similarly, Lee et al. found thinner soft palates in responders compared to nonresponders [17]. Recent work by Thuler et al. found that transverse maxillary deficiency, increased pharyngeal length, and larger relative tongue volume (tongue volume controlled by maxillomandibular volume) are associated with greater collapsibility [18, 19, 20], consistent with the observation from Isono et al. that a higher soft tissue‐to‐bony framework ratio increases airway collapsibility [21, 22]. As HGNS response has been associated with decreased airway collapsibility during DISE [13], these studies suggest that the combination of soft tissue and skeletal measurements may determine therapy outcomes. Relative tongue volume (the ratio of absolute tongue volume to total oral cavity volume) provides one quantitative measure of this soft tissue‐to‐bony framework relationship, integrating soft tissue and skeletal measurements.

The present study sought to further evaluate how CT‐derived anatomic measures are associated with the likelihood of HGNS treatment success. Our primary hypothesis was that responders would have a smaller relative tongue volume compared to nonresponders. Secondarily, we examined each component of the ratio, hypothesizing that responders to HGNS would have larger total oral cavity volumes and smaller tongue volumes compared to nonresponders. In addition to total AHI, we examined whether different associations were seen with supine versus non‐supine AHI based on previous evidence showing supine sleep position was associated with reduced effectiveness of HGNS therapy [23, 24]. An exploratory analysis was included to evaluate additional skeletal and soft tissue dimensions as markers of HGNS outcomes.

## 2 Materials and Methods

### 2.1 Participants

**FIGURE 1:**
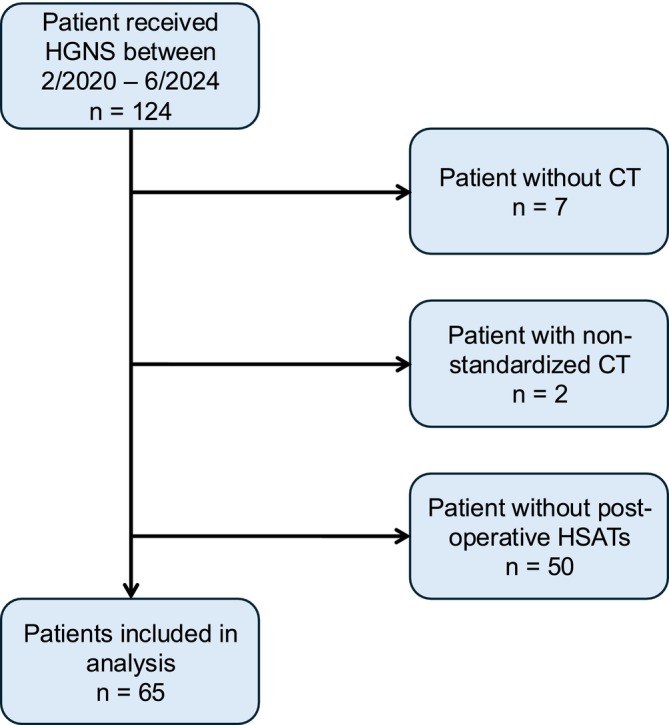
CONSORT diagram for patients included in this study. [Color figure can be viewed in the online issue, which is available at www.laryngoscope.com]

A prospective cohort study of consecutively enrolled patients seeking care from the CPAP Alternatives Clinic at the University of Pennsylvania was conducted from February 2020 until June 2024. Inclusion criteria included consenting English‐speaking adults (> 18 years) with a diagnosis of moderate to severe OSA (AHI > 15 events/h) who received HGNS implantation. All patients underwent a standard preoperative clinical assessment, including a non‐contrast facial CT and DISE to determine their eligibility for HGNS. A full‐night home sleep apnea test (HSAT) at a single therapeutic voltage was completed within 3–12 months postimplantation. Patients without a CT scan or HSAT were excluded (Figure 1). The study was approved by the Institutional Review Board at the University of Pennsylvania (IRB 850115 and 853096).

### 2.2 Study Procedures and Data Collection

#### 2.2.1 Clinical Data

Clinical data such as anthropometrics and demographics were extracted from electronic medical records. All information was stored in a secure REDCap (Nashville, TN, USA) database. Mean pharyngeal opening pressure (PhOP) and palatal opening pressure (POP) are clinical variables we collected as part of the practice. PhOP is the minimum applied positive pressure (usually via CPAP) needed to overcome pharyngeal collapse and restore airway patency [25]. POP is the level of applied PAP (cmH_2_O) during DISE at which the soft palate (velum) visibly separates from the posterior pharyngeal wall, indicating opening of the palatal airway [13].

#### 2.2.2 Sleep Study

All patients had preoperative diagnostic sleep studies (including laboratory polysomnograms [PSGs] or HSATs) within 3 years of implantation. Given diverse sources of referral, sleep study data, including total and positional (supine and non‐supine) AHI, were obtained using 3% or 4% scoring criteria. The preoperative AHI was ascertained from the most recent diagnostic sleep study before implantation. In addition, a full‐night home sleep apnea test was completed within 3–12 months postimplantation based on patient scheduling. These HSATs were administered as a postoperative efficacy study at a single setting of HGNS stimulation that was determined by the senior author to be clinically optimal and were scored primarily using a 4% desaturation criterion. When available, therapy usage was verified through Inspire Cloud (Inspire Medical Systems) software, which allows for remote monitoring of HGNS compliance. Only patients with more than 5% of sleeping time in a specific position (supine and non‐supine) were included in the positional AHI analysis (Figure 1).

#### 2.2.3 CT Protocol: Acquisition and Analysis

CT scans were obtained using scanners from either GE (GE Medical Systems) or Siemens (Siemens Healthineers). All scans were performed following a standardized CT acquisition and analysis protocol described in previous publications [18, 25]. Raw DICOM files were uploaded to the patient's chart and subsequently downloaded in anonymized form for analysis. Volumetric measurements were performed using Amira software (Thermo Fisher Scientific) by A.W., and the skeletal measures were performed using InVivo by L.C.M. and E.T. [26]. All examiners had experience with these techniques and were trained in performing the measurements. Volumetric measurements using standardized techniques have been published in prior MRI‐ and CT‐based studies [19, 27, 28], and reproducibility of skeletal measurements has been demonstrated with moderate‐to‐good interrater reliability between independent raters [29].

**FIGURE 2:**
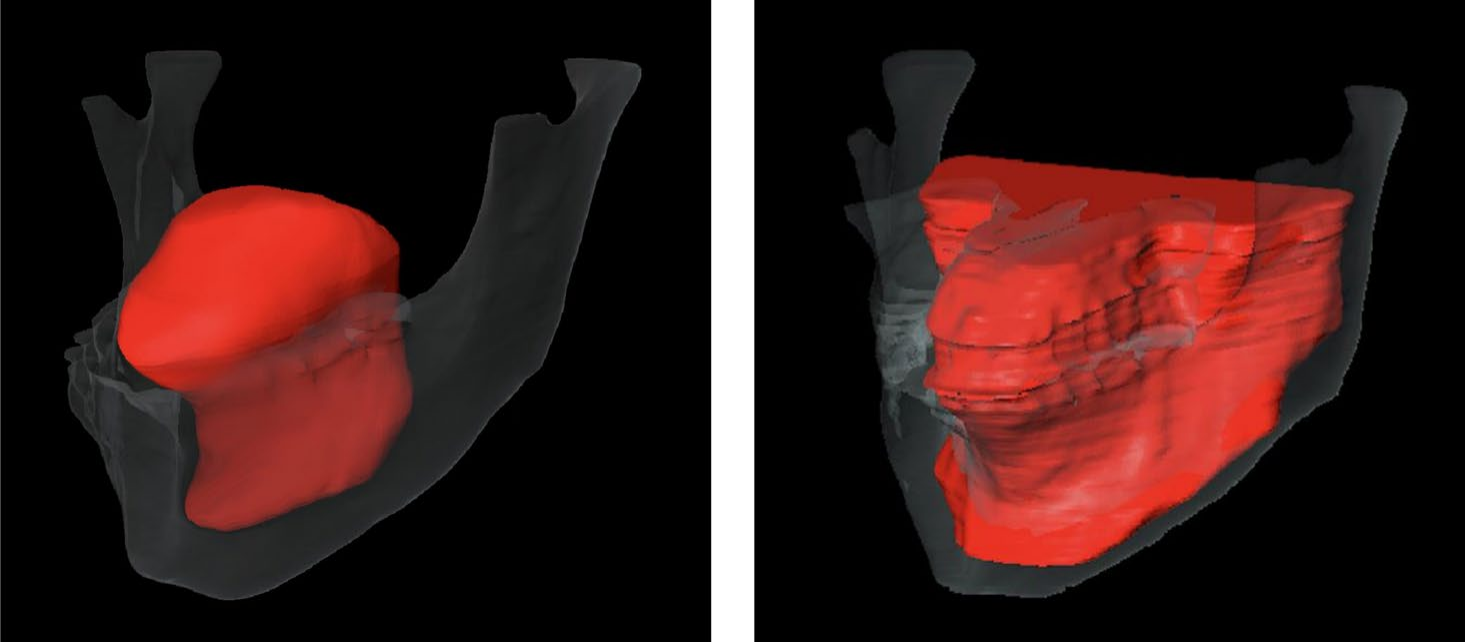
3D representations of absolute tongue volume (left) and total oral cavity volume (right). Total oral cavity volume includes intramaxillary and intramandibular volume. Main interested measurements are in red. The mandible is the translucent structure in the picture. [Color figure can be viewed in the online issue, which is available at www.laryngoscope.com]

Relative tongue volume, defined as the absolute tongue volume/total oral cavity volume, was the primary measure of interest; its individual components were secondary measures (Figure 2). Absolute tongue volume included the genioglossus and intrinsic muscles of the tongue. Total oral cavity volume was defined as the volume contained within the intraoral bounds of the maxilla and the mandible. An additional 43 measurements of soft tissues (extrinsic tongue muscles, soft palate volume, pharyngeal fat pads), airway size (cross‐sectional areas in the retroglossal and retropalatal regions), and skeletal measurements (linear distances and areas) were examined in the exploratory analysis. For a complete list of measurements, see Table S1.

### 2.3 Statistical Analysis

Treatment response was evaluated based on the Sher criteria using total AHI (primary) and positional (supine, non‐supine) AHI (secondary). Sher's criteria define responders as patients who have > 50% reduction in AHI and a postoperative AHI of < 20 events/h [3]. Categorical variables are summarized as frequencies and percentages, while continuous variables are summarized using means and standard deviations (SDs). Where applicable, changes (absolute or percent) were defined as postoperative minus preoperative values, with percent change expressed relative to the preoperative value.

For unadjusted comparisons of responders and nonresponders, Student's *t*‐tests were used for continuous variables and chi‐squared tests were used for categorical variables. Logistic regression models were used to evaluate associations between anatomical and clinical covariates with HGNS response, both unadjusted and adjusted for age, sex, and BMI. Results of logistic regression analyses are reported using standardized odds ratios (ORs; equal to the relative change in odds of being a responder associated with a 1 SD increase in the independent variable), along with associated 95% confidence intervals (CIs) and *p* values. In addition, for descriptive purposes, group‐specific adjusted means and 95% CIs for continuous anatomical measures were estimated from linear regression models with covariate adjustment (age, sex, and BMI).

For sensitivity analysis, assessments were repeated after excluding four patients with a high frequency of central sleep apnea events (> 5 events/h) on their postoperative sleep study. Newly developed postoperative central AHI > 5 events/h was a phenomenon previously described in HGNS implantation (treatment‐emergent central sleep apnea, or TECSA) [30].

For primary analyses examining the association of relative tongue volume with responder status, a *p* < 0.05 was considered statistically significant. All analyses were conducted using Stata/SE Version 14.2 or higher (StataCorp, College Station, TX, USA).

## 3 Results

### 3.1 Sample Characteristics

A total of 65 patients met the study inclusion criteria. There were 31 responders and 34 nonresponders based on the Sher criteria using total AHI. On average, patients were 63.3 ± 11.7 years old, overweight (BMI: 29.5 ± 4.0 kg/m^2^), about half were male (50.8%), and most were White (89.2%). Participants had moderate to severe AHI on average (28.6 ± 13.3 events/h). Mean PhOP was 6.1 ± 2.1 cmH_2_O, and mean POP was 5.8 ± 2.0 cmH_2_O. There were no statistically significant differences in demographics between responders and nonresponders (Table 1).

**TABLE 1:** Sample characteristics.

Characteristic	Total (*n* = 65)	Responders (*n* = 31)	Nonresponders (*n* = 34)	*p*
Age (years)	63.3 ± 11.7	62.3 ± 12.8	64.2 ± 10.7	0.50
Male sex (%)	50.8	45.2	55.9	0.39
Body mass index (kg/m^2^)	29.5 ± 4.0	28.8 ± 3.9	30.1 ± 4.0	0.18
White (%)	89.2	83.9	94.1	0.14
Preoperative AHI (events/h)				
Total	28.6 ± 13.3	27.8 ± 15.1	29.4 ± 11.4	0.62
Supine	40.5 ± 17.6	36.4 ± 18.3	47.7 ± 16.6	0.07
Non‐supine	17.6 ± 13.6	17.6 ± 13.5	17.5 ± 14.0	0.63
Pharyngeal opening pressure (PhOP) (cmH_2_O)	6.1 ± 2.1	6.2 ± 2.2	6.1 ± 2.2	0.89
Palatal opening pressure (POP) (cmH_2_O)	5.8 ± 2.0	5.8 ± 2.0	5.8 ± 2.0	0.99

### 3.2 Association of Relative Tongue Volume With HGNS Response

In primary analyses adjusted for clinical covariates (Table 2), relative tongue volume was not significantly associated with the likelihood of HGNS response (OR [95% CI] = 0.82 [0.48, 1.40]; *p* = 0.462). The adjusted mean (95% CI) relative tongue volume was 0.50 (0.47, 0.53) in responders and 0.52 (0.49, 0.54) in nonresponders. Similarly, there was no significant relationship when analyzing response status using supine and non‐supine AHI (Table 2). Unadjusted analyses are shown in Table S2. In the sensitivity analysis excluding patients with significant postoperative central sleep apnea, results were similar (Table S3).

**TABLE 2:** Association of relative tongue volume and its components with HGNS response defined by total and positional AHI.

Measures	AHI type (nR/nNR)	Adjusted mean (95% CI)^a^		OR (95% CI)^a^, ^b^	*p*
		Responders	Nonresponders		
Relative tongue volume	Total (30/33)	0.50 (0.47, 0.53)	0.52 (0.49, 0.54)	0.82 (0.48, 1.40)	0.462
	Supine (22/29)	0.53 (0.50, 0.56)	0.51 (0.48, 0.54)	1.35 (0.71, 2.57)	0.355
	Non‐supine (21/24)	0.50 (0.47, 0.54)	0.54 (0.51, 0.58)	0.58 (0.29, 1.15)	0.117
Absolute tongue volume (mm^3^)	Total (30/34)	90,990 (85,989, 95,991)	88,772 (84,082, 93,463)	1.27 (0.62, 2.61)	0.518
	Supine (22/29)	89,750 (83,750, 95,751)	90,348 (85,145, 95,552)	0.93 (0.42, 2.09)	0.867
	Non‐supine (21/25)	91,041 (84,725, 97,357)	91,210 (85,456, 96,963)	0.93 (0.37, 2.33)	0.873
Total oral cavity volume (mm^3^)	Total (30/33)	168,364 (158,464, 178,265)	158,096 (148,666, 167,526)	1.81 (0.82, 3.99)	0.139
	Supine (22/29)	157,694 (145,948, 169,439)	162,308 (152,124, 172,492)	0.75 (0.32, 1.77)	0.518
	Non‐supine (21/24)	166,522 (153,664, 179,380)	156,025 (144,049, 168,001)	1.71 (0.61, 4.81)	0.312

Abbreviations: nNR, number of nonresponders; nR, number of responders.

^a^ Models adjusted for age, sex, and BMI.

^b^ Standardized OR equal to the relative change in odds of being a responder for a 1 SD increase in anatomy measure.

### 3.3 Association of Tongue and Oral Cavity Volumes With HGNS Response

In secondary analyses (Table 2), neither absolute tongue volume (OR [95% CI] = 1.27 [0.62, 2.61]; *p* = 0.518) nor total oral cavity volume (OR [95% CI] = 1.81 [0.82, 3.99]; *p* = 0.139) was significantly associated with HGNS response. After covariate adjustment, mean (95% CI) tongue volume was 90,990 (85,989, 95,991) mm^3^ in responders and 88,772 (84,082, 93,463) mm^3^ in nonresponders. The total oral cavity volume was 168,364 (158,464, 178,265) mm^3^ in responders and 158,096 (148,666, 167,526) mm^3^ in nonresponders. The relationship remained nonsignificant when analyzing response status using supine or non‐supine AHI.

### 3.4 Exploratory Analysis

An additional 43 measurements were classified into anatomical domains, including soft tissue, airway size, cephalometric parameters, maxillomandibular dimensions, and hyoid position. Of these, the hyoid‐to‐mandible distance in the hyoid position domain, the transverse maxillary dimensions (transverse maxillary area and intermolar distance) in the maxillomandibular domain, and epiglottis volume remained significant after covariate adjustment (Table S4).

#### 3.4.1 Hyoid Position

**FIGURE 3:**
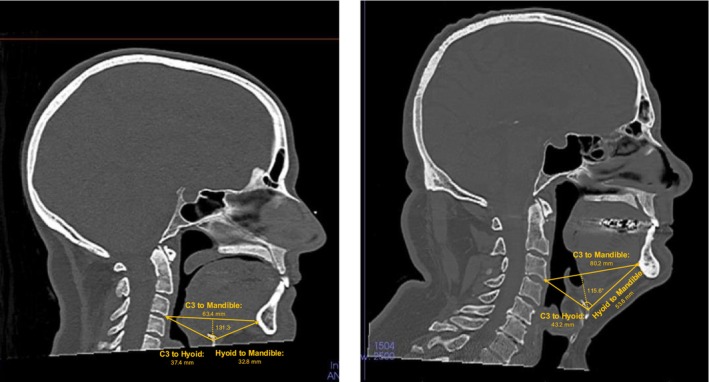
Examples of hyoid position in a representative responder (left) and nonresponder (right). Solid lines indicate linear distances between C3, hyoid, and mandible, including C3‐hyoid, hyoid‐mandible, and C3‐mandible distances. Dashed lines are the perpendicular distance from the hyoid to the C3‐mandibular plane. The angle formed by C3‐hyoid‐mandible is also shown. [Color figure can be viewed in the online issue, which is available at www.laryngoscope.com]

Hyoid‐related measurements included distance from hyoid‐to‐mandibular plane (C3‐Msy), C3 vertebrae‐hyoid‐mandible angle (C3hm), and distance from hyoid to mandible (hm) (Figure 3). The hyoid‐to‐mandible distance was significantly associated with the response status (OR [95% CI] = 0.46 [0.25, 0.86]; *p* = 0.015). Responders had a smaller distance compared with nonresponders (41.7 [39.3, 44.0] mm vs. 45.9 [43.7, 48.2] mm). Other hyoid metrics were not significant. On average, responders had some evidence of a shorter hyoid‐to‐mandibular plane distance (8.4 [5.8, 10.9] vs. 11.8 [9.4, 14.2] mm; OR [95% CI] = 0.55 [0.30, 1.02]; *p* = 0.059).

#### 3.4.2 Transverse Maxillary Dimensions

**FIGURE 4:**
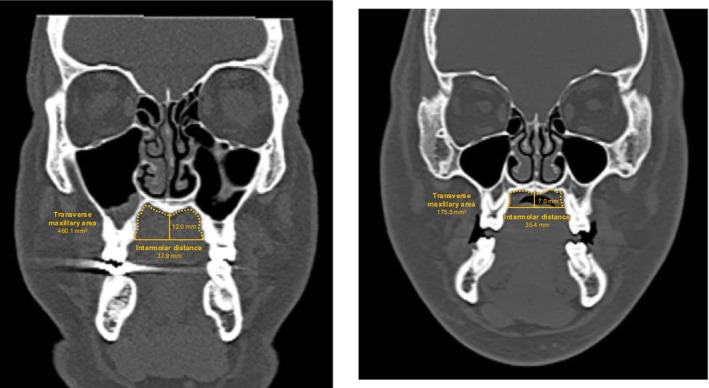
Examples of transverse maxillary dimensions in a representative responder (left) and nonresponder (right). Solid lines depict the linear distance between the maxillary first molars and the intermolar height, defined as the perpendicular line from the intermolar distance to the palate. The transverse maxillary area is outlined by dashed lines. [Color figure can be viewed in the online issue, which is available at www.laryngoscope.com]

Measurements in the maxillomandibular domain at the level of the first molar of the maxilla included intermolar distance, intermolar height, and transverse maxillary area (or intermolar area in the upper jaw) (Figure 4). The transverse maxillary area and intermolar distance were significantly associated with the response status. Responders had a larger transverse maxillary area compared to nonresponders (337.6 [306.0, 369.2] vs. 266.2 [239.4, 293.1] mm; OR [95% CI] = 2.79 [1.42, 5.49]; *p* = 0.003). On average, responders had larger intermolar distance (32.3 [31.0, 33.6] vs. 30.3 [29.2, 31.4] mm; OR [95% CI] = 2.18 [1.07, 4.43]; *p* = 0.032). Intermolar height was not significantly associated with response status.

#### 3.4.3 Epiglottis Volume

The epiglottis volume was significantly associated with the response status. Responders had larger epiglottis volume compared to nonresponders (1222.8 [986.2, 1459.5] vs. 877.5 [655.5, 1099.4] mm^3^; OR [95% CI] = 1.93 [1.02, 3.66]; *p* = 0.044).

## 4 Discussion

This is the most comprehensive study to date examining soft tissue and skeletal measurements in a patient population who received HGNS implants, using standardized CT scan acquisition and analysis. Contrary to our initial hypothesis, the relative tongue volume and its components were not significantly associated with HGNS response defined by total, supine, and non‐supine AHI. In the exploratory analysis, we observed associations related to maxillary dimensions, hyoid position, and epiglottis volume; though these findings should be interpreted cautiously and considered hypothesis‐generating, given the limited sample size and exploratory nature of the analysis.

This work expands on previous research demonstrating the possible utility of CT imaging in assessing airway collapsibility [18, 19, 25]. Thuler et al. found that craniofacial anatomy is associated with increased airway collapsibility [18, 19, 20]. While larger absolute tongue volumes were associated with increased collapsibility, the relative tongue volume has not been evaluated [19]. In our study, the association between relative tongue volume and HGNS response was not significant. Prior studies have modeled the pharynx as a collapsible tube within a rigid chamber, reflecting how soft tissues are enclosed by bony structures such as the mandible, maxilla, and cervical spine [22, 31]. While this model is intuitively appealing, our findings suggest that it alone does not fully account for the pathophysiology of OSA or the variability in response to HGNS. Other factors, including suboptimal voltage, incorrect electrode configuration, and neurophysiological factors, likely contribute to suboptimal response to HGNS [32, 33, 34]. Recognizing these nonanatomic contributors highlights that HGNS outcomes cannot be explained by imaging alone and require a broader, integrative framework.

Absolute tongue volume and total oral cavity volume were also not significantly associated with HGNS response, defined by total, supine, and non‐supine AHI. Previous anatomic studies showed that apneic patients had higher tongue fat disposition than controls [35, 36, 37]. With a small sample size, Schwab et al. found no significant relationship between tongue volumes and response status [16]. Similarly, we did not find a significant relationship between tongue volume and HGNS response. Although not statistically significant, responders had smaller relative tongue volume than nonresponders, largely due to greater oral cavity volume. Larger studies are needed to clarify these relationships. For example, to maintain > 80% power to detect an OR of 1.81 (as observed for oral cavity volume in our data), a future study would require 50 responders and 50 nonresponders, whereas detecting an OR of 0.82 (relative tongue volume) would require nearly 400 per group. While 100 patients are feasible in a single study, larger cohorts may necessitate incorporating these measures into multisite HGNS registries.

Positional outcomes were evaluated in our primary and secondary hypotheses, given the clinical relevance of HGNS in positional OSA [23, 38]. In our cohort, neither relative tongue volume nor its component measures were associated with treatment response when outcomes were defined using supine or non‐supine AHI. These findings are consistent with the lack of association observed using total AHI and suggest that the relationship between craniofacial anatomy and HGNS efficacy does not differ meaningfully across sleeping positions. These results should be interpreted with caution, as the positional analyses had reduced statistical power due to some participants without sufficient sleep time in each position.

Exploratory analyses revealed a relationship between treatment response and hyoid position, maxillary dimension, and epiglottic volume. Only the hyoid‐to‐mandible distance was significantly associated with response, but average values of other metrics were also consistent with responders having a more superiorly positioned hyoid, reflected by shorter hyoid‐mandibular and hyoid‐mandibular plane distances and a wider C3‐hyoid‐mandible angle (Figure 3). This aligns with prior literature linking inferior and posterior hyoid positioning with increased upper airway collapsibility and greater OSA severity [39, 40, 41, 42, 43]. Although hyoid position can influence airway collapsibility through several established mechanisms, PhOP and POP were comparable between responders and nonresponders in our cohort, suggesting that collapsibility differences alone do not account for the association observed in our study [13, 44]. Our results suggest that a low hyoid may create an unfavorable vector for tongue motion during stimulation, as genioglossus and related muscles produce less forward and more vertical tongue movement. During sleep, the tongue must overcome not only the passive collapsibility of the pharynx but also the negative effort dependence of the upper airway, which tends to draw the tongue backward [45, 46]. Therefore, effective forward tongue motion is critical to maintaining airway patency. Previous studies have demonstrated that when tongue motion is directed both anteriorly and vertically, rather than predominantly forward, pharyngeal patency is reduced, underscoring the importance of the vector of tongue movement in upper airway mechanics [47].

In addition, previous studies have assessed transverse dimension deficiency using measures such as interpremolar and intermolar distances, as well as intramaxillary and intramandibular volumes, with smaller dimensions associated with increased airway collapsibility [18, 19, 20]. More recently, Weiner et al. reported that supine pharyngeal width, another transverse anatomy surrogate, was associated with response to HGNS, with responders having larger transverse dimensions, consistent with the results from this study [48]. In our study, both the intermolar distance and transverse maxillary area were associated with being a responder. The transverse maxillary area assesses skeletal deficiency in two dimensions overlying the tongue dorsum (Figure 4). While the hyoid position determines tongue orientation, the maxillary area determines the amount of space for tongue motion. Reduced maxillary area will restrict the displacement of the hydrostatic tongue, limiting the degree of pharyngeal airway opening. In contrast, and contrary to our expectation, responders had larger epiglottis volume compared to nonresponders in our analysis; future studies are required to verify this unexpected association. Our hypothesis‐generating principles are that hyoid position affects the tongue's vector while the maxillary area determines the tongue's displacement, both potentially important factors in the HGNS effectiveness.

The study had several strengths. We analyzed a comprehensive set of skeletal and soft tissue measurements in a relatively large patient population compared to previous studies. A common limitation in imaging‐based studies is inconsistency in image acquisition and analysis [17]. To address this, we used a standardized approach to obtain and analyze CT scans, a method that has been replicated in prior studies [18, 25]. In addition, we used HSATs exclusively to extract postoperative AHI, aiming to capture sleep in a natural environment and more accurately assess response to HGNS. This approach may result in lower observed response rates compared to titration PSGs, which often capture data across various device settings that may not reflect a typical night's sleep [49].

We also acknowledge several limitations of this study. First, this was a single‐center cohort clinically selected according to the senior author's criteria for HGNS candidacy, which may limit generalizability. Second, in some patients, the AHI was derived preoperatively from PSGs and postoperatively from HSATs, which differ in signal acquisition and hypopnea scoring. PSGs may include arousal‐based hypopneas and use either 3% or 4% desaturation thresholds, whereas postoperative HSATs were primarily scored using a 4% criterion and do not capture arousal‐only events, potentially underestimating hypopneas and introducing variability in pre–post comparisons [50, 51]. After reviewing our data, we identified mismatched pre‐ and postoperative hypopnea criteria in 4 of 65 patients (6%). To assess whether these differences meaningfully influenced outcomes, we compared responder rates across testing modalities and found that patients who underwent both baseline and follow‐up HSAT had a lower responder rate (41%) compared with those who had baseline PSG and follow‐up HSAT (53%); however, this difference was not statistically significant (*p* = 0.34). Given that hypopnea mismatches occurred in both directions and affected a small proportion of the cohort, substantial bias is unlikely, although AHI differences should be interpreted with caution. Third, some postoperative sleep studies were interpreted by the senior author as part of routine clinical care. Although scoring followed standardized protocols and was independent of study analyses, this practice may introduce potential bias and is acknowledged as a limitation. Fourth, the awake CT protocol does not capture sleep‐related changes of jaw position and related dynamic collapse, as seen on state‐dependent MRI [52]. Accordingly, our imaging findings should be interpreted in the context of these limitations and do not support routine CT imaging for HGNS evaluation outside of surgical‐planning contexts. Finally, our exploratory analyses (i.e., hypothesis‐generation) were not corrected for multiple hypothesis testing as they are designed to be explicitly tested in an independent sample.

## 5 Conclusion

Our primary and secondary analyses did not identify statistically significant associations between relative tongue volume or its individual components and HGNS response using total or positional AHI, suggesting nonanatomic factors may be more important. Exploratory analyses suggest that anatomical factors (e.g., lower hyoid position or larger maxillary dimensions) could play a role in HGNS outcomes. Future studies with hyoid‐related primary outcomes in a larger data set may provide greater insight into anatomical markers of HGNS response.

## Funding

This study was made possible by a grant from the National Institute of Health (1R01HL144859).

## Conflicts of Interest

Eric Thuler reports receiving grant research funding from NIH and Inspire. Brendan T. Keenan reports receiving a salary for consulting work with Biomedical Statistical Consulting LLC. Richard J. Schwab reports receiving grant research funding from NIH. Raj C. Dedhia reports receiving grant research funding from NIH, Inspire, Cryosa, and Nyxoah Medical, and serving on the clinical advisory board for Lunair Medical. The other authors declare no conflicts of interest.

## Supporting information


**Table S1:** Description of soft tissue, skeletal, and airway measurements.
**Table S2:** Unadjusted analysis of relative tongue volume and its components between responders and nonresponders defined by total and positional AHI.
**Table S3:** Adjusted sensitivity analysis of relative tongue volume and its components between responders and nonresponders defined by total and positional AHI.
**Table S4:** Adjusted exploratory analysis between responders and nonresponders defined by total and positional AHI.

## Data Availability

The data that support the findings of this study are available on request from the corresponding author. The data are not publicly available due to privacy or ethical restrictions.
