# Supplementary material for: The Association of Upper Airway Anatomy and Hypoglossal Nerve Stimulation Response in OSA
Source: Laryngoscope. 2026 Mar 19;136(8):3630–8. doi: 10.1002/lary.70493 (PMC13357322; doi:10.1002/lary.70493)
Supplement: Supplementary file 1 — Table S1: Description of soft tissue, skeletal, and airway measurements. Table S2: Unadjusted analysis of relative tongue volume and its components between responders and nonresponders defined by total and positional AHI. Table S3: Adjusted sensitivity analysis of relative tongue volume and its components between responders and nonresponders defined by total and positional AHI. Table S4: Adjusted exploratory analysis between responders and nonresponders defined by total and positional AHI. [file LARY-136-3630-s001.docx]

**Table S1.** Description of soft tissue, skeletal, and airway measurements

| **Variables** | **Description** | **Abbreviation** |
| --- | --- | --- |
| Retropalatal airway volume  (mm^3^) | The airway appearing on the same axial slices as the soft palate | rpa |
| Retroglossal airway volume  (mm^3^) | Retroglossal, all airway not appearing on the same axial slices as the soft palate | rga |
| Number of slices in the RP region | number of slices in the RP region | rpslice |
| Number of slices in the RG region | number of slices in the RG region | rgslice |
| Epiglottis volume (mm^3^) | Epiglottis volume in full | epi1 |
| Soft palate volume (mm^3^) | Soft palate volume including the uvula | sp |
| Absolute tongue volume (mm^3^) | Genioglossus plus intrinsic muscles | t |
| Extrinsic tongue muscles except genioglossus volume (mm^3^) | All extrinsic tongue muscles other than the genioglossus | ot |
| Medial pterygoids volume (mm^3^) | The medial pterygoid muscles, segmented from attachment at mandible to the limit of region of interest | pt |
| Parapharyngeal fat pads volume (mm^3^) | The parapharyngeal fat pads, located between the pterygoids and the lateral walls of the pharynx | fp |
| Retropalatal lateral walls volume (mm^3^) | The lateral and posterior pharyngeal walls on the same axial slices as the soft palate | rplw |
| Retroglossal lateral walls volume (mm^2^) | The lateral and posterior pharyngeal walls NOT on the same axial slices as the soft palate. | rglw |
| Minimum cross-sectional area of retropalatal area (mm^2^) | Minimum cross-sectional area of retropalatal area | rpminarea |
| Minimum cross-sectional area of retroglossal area (mm^2^) | Minimum cross-sectional area of retroglossal area | rgminarea |
| Intramaxillary volume (mm^3^) | Volume of space within maxillary oral cavity, bounded inferiorly by alveolar ridge | imxv |
| Mandibular volume in retropalatal region (mm^3^) | Volume of space within bounds of mandible, in retropalatal region | rpimv |
| Mandibular volume in retroglossal region (mm^3^) | Volume of space within bounds of mandible, in retroglossal region | rgimv |
| Extended mandibular volume in retropalatal region (mm^3^) | Mandibular volume in retropalatal region rectangularly extended back to anterior edge of spine | rpimve |
| Extended mandibular volume in retroglossal region (mm^3^) | Extended mandibular volume in retroglossal region rectangularly extended back to anterior edge of spine | rgimve |
| Total oral cavity volume (mm^3^) | Intramaxillary and intramandibular volume | imvtotal |
| Extended total oral cavity volume (mm^3^) | Total oral cavity volume rectangularly extended back to anterior edge of spine | imvetotal |
| Relative tongue volume (mm^3^) | Absolute tongue volume/ total oral cavity volume | relative_t |
| Head anteriorization (C4pi-sella-nasion angle) (degree) | Angle measured between the most anterior-inferior aspect of the fourth cervical vertebrae (C4), Sella and Nasion | anteriorization |
| Head extension (Fmp-atlas plane angle) (degree) | Measured by the angle between the posterior rim of foramen magnum and the plane determined by the most posterior and the most anterior aspect of atlas bone (first cervical vertebrae – C1) | extension |
| SNA (degree) | Angle formed by the Sella, Nasion and the A point below the anterior nasal spine | sna |
| Nasion A distance (mm) | Distance from Nasion to A point at the maxilla (Upper facial height) | nasiona |
| SNB (degree) | Angle formed by the Sella, Nasion and B point at the mandible (most posterior point between the chin and mandibular incisors) | snb |
| Nasion to B point distance (mm) | Distance from nasion to B point at the mandible (Facial height) | nasionb |
| Cranial base angle (degree) | Angle between nasion, Sella, and Basion | bsa |
| Posterior cranial base (mm) | Distance from Sella to Basion | pcb |
| Anterior cranial base (mm) | Distance from Nasion to Sella | acb |
| Hard palate length (mm) | Distance from anterior nasal spine (ANS) to posterior nasal spine (PNS) | hpl |
| PNS to hyoid distance (mm) | Distance between the posterior nasal spine and the body of hyoid bone | pnh |
| Distance Hyoid to Mandibular plane (mm) | Perpendicular distance from hyoid to the plane connecting cervical vertebrae (C3) to posterior-inferior aspect of mandibular symphysis | c3-msy |
| C3-Hyoid-Mandible angle (degree) | Angle formed between C3 - hyoid body - mandibular symphysis | c3hm |
| Distance C3 to Hyoid body (mm) | Distance between third cervical vertebrae (C3) and hyoid body | c3hb |
| Distance Hyoid to Mandible (mm) | Distance between hyoid and posterior-inferior aspect of mandibular symphysis | hm |
| Interpremolar distance at interalveolar ridge upper jaw (mm) | Distance between the first premolars of upper jaw | ipmdu |
| Interpremolar height upper jaw (mm) | Distance from interpremolar plane to the hard palate | ipm height |
| Interpremolar Area upper jaw (mm^2^) | Area bounded interpremolar plane and hard palate of upper jaw | ipm area |
| Interpremolar distance at interalveolar ridge lower jaw (mm) | Distance between the first premolars of lower jaw | ipmdl |
| Intermolar distance at interalveolar ridge upper jaw (mm) | Distance between the first molars of upper jaw | imdu |
| Intermolar height upper jaw (mm) | Distance from intermolar plane to the hard palate | imd height |
| Intermolar area upper jaw (mm^2^) | Area bounded intermolar plane and hard palate of upper jaw | imau |
| Intermolar distance lingual alveolar ridge lower jaw (mm) | Distance between the first molars of lower jaw | imdl |
| Upper Jaw axial area (mm^2^) | Maxillary intra-oral area, measures at the level of the lingual alveolar bone in axial view | ujaa |

**Table S2.** Unadjusted analysis of relative tongue volume and its components between responders and non-responders defined by total and positional AHI

| **Measurements** | **AHI type**  **(nR/nNR)** | **Responders** | **Nonresponders** | **p-value** | **Odds ratios (95% CI)** | **p-value (OR)** |
| --- | --- | --- | --- | --- | --- | --- |
| Relative tongue volume  mean ± SD | Total  (30/33) | 0.50 ± 0.07 | 0.52 ± 0.09 | 0.402 | 0.80  (0.48-1.33) | 0.396 |
|  | Supine  (22/29) | 0.53 ± 0.09 | 0.51 ± 0.07 | 0.418 | 1.27  (0.71-2.27) | 0.411 |
|  | Nonsupine  (21/24) | 0.50 ± 0.06 | 0.54 ± 0.09 | 0.110 | 0.60  (0.32-1.13) | 0.114 |
| Absolute tongue volume (mm^3^)  mean ± SD | Total  (30/34) | 88913 ± 16483 | 90605 ± 20133 | 0.716 | 0.91  (0.55-1.50) | 0.711 |
|  | Supine  (22/29) | 85829 ± 13867 | 93323 ± 21922 | 0.167 | 0.67  (0.38-1.19) | 0.168 |
|  | Nonsupine  (21/25) | 87260 ± 18220 | 94286 ± 19271 | 0.207 | 0.67  (0.37-1.24) | 0.206 |
| Total oral cavity volume (mm^3^)  mean ± SD | Total  (30/33) | 165241 ± 38494 | 160935 ± 37020 | 0.653 | 1.13  (0.68-1.86) | 0.647 |
|  | Supine  (22/29) | 150462 ± 32270 | 167794 ± 40595 | 0.106 | 0.61  (0.34-1.12) | 0.112 |
|  | Nonsupine  (21/24) | 159645 ± 37748 | 162043 ± 39497 | 0.837 | 0.94  (0.51-1.71) | 0.832 |
| nR: number of responders; nNR: number of non-responders | | | | | | |

**Table S3.** Adjusted sensitivity analysis of relative tongue volume and its components between responders and nonresponders defined by total and positional AHI

| **Measures** | **AHI type**  **(nR/nNR)** | **Adjusted Mean (95% CI)*** | | **OR**  **(95% CI)*^, †^** | **p-value** |
| --- | --- | --- | --- | --- | --- |
|  |  | **Responders** | **Non-responders** |  |  |
| Relative tongue volume | Total  (29/30) | 0.50  (0.47, 0.53) | 0.52  (0.49, 0.55) | 0.75 (0.43, 1.31) | 0.313 |
|  | Supine  (21/26) | 0.53  (0.50, 0.57) | 0.52  (0.49, 0.55) | 1.23 (0.64, 2.38) | 0.529 |
|  | Non-supine  (21/24) | 0.50  (0.46, 0.54) | 0.56  (0.52, 0.59) | 0.45 (0.21, 0.95) | 0.038 |
| Absolute tongue volume (mm^3^) | Total  (29/31) | 90984  (85881, 96086) | 89406  (84474, 94337) | 1.18 (0.57, 2.41) | 0. 658 |
|  | Supine  (21/26) | 89621  (83439, 95804) | 91335  (85795, 96875 | 0.83 (0.37, 1.86) | 0.656 |
|  | Non-supine  (21/22) | 89938  (83729, 96146) | 92625  (86571, 98678) | 0.66 (0.25, 1.72) | 0.395 |
| Total oral cavity volume (mm^3^) | Total  (29/30) | 168327  (158146, 178507) | 157328  (147322, 167334) | 1.85 (0.84, 4.09) | 0.127 |
|  | Supine  (21/26) | 157017  (144787, 169247) | 162002  (151044, 172960) | 0.75 (0.32, 1.77) | 0.505 |
|  | Non-supine  (21/21) | 224623  (208453, 240792) | 211077  (194908, 227247) | 1.71 (0.61, 4.81) | 0.312 |
| nR: number of responders; nNR: number of non-responders; *Models adjusted for age, sex and BMI; ^†^Standardized OR equal to the relative change in odds of being a responder for a 1 SD increase in anatomy measure | | | | | |

**Table S4.** Adjusted exploratory analysis between responders and nonresponders defined by total AHI

| **Measures** | **Domain** | **Adjusted Mean (95% CI)*** | | **OR**  **(95% CI)*^, †^** | **p-value** |
| --- | --- | --- | --- | --- | --- |
|  |  | **Responders**  **(n=30)** | **Non-responders**  **(n=34)** |  |  |
| epi1 | Soft tissue | 1222.8  (986.2, 1459.5) | 877.5  (655.5, 1099.4) | 1.93 (1.02, 3.66) | 0.044^**^ |
| sp | Soft tissue | 8752.8  (7943.9, 9561.8) | 8621.7  (7863.0, 9380.4) | 1.08 (0.58, 2.03) | 0.804 |
| ot | Soft tissue | 32836.5  (29954.4, 35718.5) | 33669.1  (30966.1, 36372.2) | 0.86 (0.45, 1.65) | 0.658 |
| pt | Soft tissue | 16200.3  (14977.0, 17423.7) | 15217.4  (14070.0, 16364.7) | 1.54 (0.75, 3.18) | 0.242 |
| fp | Soft tissue | 5079.9  (4543.4, 5616.4) | 5322.3  (4819.1, 5825.5) | 0.83 (0.47, 1.45) | 0.508 |
| rplw | Soft tissue | 12606.3  (11519.2, 13693.5) | 11670.7  (10651.2, 12690.3) | 1.50 (0.79, 2.84) | 0.214 |
| rglw | Soft tissue | 12156.5  (10842.7, 13470.4) | 11819.6  (10587.4, 13051.8) | 1.13 (0.61, 2.07) | 0.702 |
| rpa | Airway size | 5842.7  (4853.1, 6832.4) | 6475.7  (5547.6, 7403.9) | 0.73 (0.39, 1.37) | 0.330 |
| rga | Airway size | 9672.3  (7634.6, 11709.9) | 9717.1  (7806.0, 11628.2) | 0.98 (0.55, 1.75) | 0.945 |
| rpslice | Airway size | 10.3  (9.5, 11.1) | 10.3  (9.6, 11.1) | 0.98 (0.55, 1.75) | 0.949 |
| rpminarea | Airway size | 113.4  (88.2, 138.5) | 120.3  (96.6, 143.9) | 0.89 (0.52, 1.53) | 0.669 |
| rgslice | Airway size | 11.4  (10.4, 12.3) | 11.7  (10.8, 12.6) | 0.88 (0.52, 1.46) | 0.612 |
| rgminarea | Airway size | 211.0  (168.9, 253.2) | 193.4  (153.9, 233.0) | 1.20 (0.67, 2.16) | 0.544 |
| anteriorization | Cephalometrics | 114.0  (111.8, 116.2) | 115.6  (113.5, 117.8) | 0.74 (0.43, 1.28) | 0.282 |
| extension | Cephalometrics | 18.4  (17.0, 19.8) | 16.9  (15.6, 18.3) | 1.60 (0.87, 2.94) | 0.130 |
| sna | Cephalometrics | 86.1  (84.6, 87.5) | 85.3  (83.9, 86.6) | 1.27 (0.72, 2.27) | 0.408 |
| nasiona | Cephalometrics | 53.3  (52.0, 54.6) | 51.6  (50.4, 52.9) | 1.85 (0.96, 3.58) | 0.067 |
| snb | Cephalometrics | 82.2  (80.8, 83.5) | 81.7  (80.5, 83.0) | 1.16 (0.65, 2.07) | 0.608 |
| nasionb | Cephalometrics | 95.2  (92.3, 98.0) | 93.3  (90.7, 96.0) | 1.37 (0.71, 2.62) | 0.346 |
| bsa | Cephalometrics | 123.6  (121.3, 126.0) | 124.2  (122.0, 126.4) | 0.90 (0.51, 1.60) | 0.724 |
| pcb | Cephalometrics | 46.2  (44.8, 47.7) | 44.4  (43.0, 45.7) | 1.88 (0.95, 3.72) | 0.072 |
| acb | Cephalometrics | 70.9  (69.7, 72.2) | 70.8  (69.6, 72.0) | 1.04 (0.55, 1.95) | 0.908 |
| hpl | Cephalometrics | 53.0  (51.6, 54.3) | 53.0  (51.7, 54.3) | 0.97 (0.51, 1.88) | 0.938 |
| imxv | Maxillomandibular | 15552.2  (14238.1, 16866.3) | 14792.0  (13540.3, 16043.6) | 1.30 (0.71, 2.37) | 0.398 |
| rpimv | Maxillomandibular | 113547.1  (102180.3, 124913.8) | 111088.3  (100262.0, 121914.6) | 1.11 (0.58, 2.12) | 0.754 |
| rgimv | Maxillomandibular | 54817.3  (46763.2, 62871.4) | 47007.7  (39336.6, 54678.9) | 1.50 (0.84, 2.66) | 0.168 |
| rpimve | Maxillomandibular | 123819.9  (110881.9, 136757.9) | 123288.1  (110965.2, 135610.9) | 1.02 (0.55, 1.89) | 0.953 |
| rgimve | Maxillomandibular | 101001.6  (89381.2, 112622.0) | 89576.7  (78508.8, 100644.6) | 1.50 (0.85, 2.64) | 0.157 |
| imvetotal | Maxillomandibular | 224821.5  (213231.2, 236411.8) | 212864.7  (201825.5, 223904.0) | 1.75 (0.83, 3.69) | 0.142 |
| ipmdu | Maxillomandibular | 27.3  (26.2, 28.4) | 26.8  (25.8, 27.8) | 1.22 (0.68, 2.20) | 0.502 |
| ipmhu | Maxillomandibular | 7.9  (6.9, 9.0) | 7.6  (6.7, 8.6) | 1.11 (0.65, 1.90) | 0.695 |
| ipmau | Maxillomandibular | 200.4  (171.0, 229.8) | 193.6  (167.6, 219.6) | 1.10 (0.62, 1.92) | 0.751 |
| ipmdl | Maxillomandibular | 26.9  (25.4, 28.4) | 26.2  (24.9, 27.6) | 1.20 (0.68, 2.10) | 0.526 |
| imdu | Maxillomandibular | 32.3  (31.0, 33.6) | 30.3  (29.2, 31.4) | 2.18 (1.07, 4.43) | 0.032^**^ |
| imhu | Maxillomandibular | 9.9  (8.8, 11.0) | 9.0  (8.1, 9.9) | 1.48 (0.81, 2.69) | 0.202 |
| imau | Maxillomandibular | 337.6  (306.0, 369.2) | 266.2  (239.4, 293.1) | 2.79 (1.42, 5.49) | 0.003^**^ |
| imdl | Maxillomandibular | 35.4  (33.3, 37.4) | 34.0  (32.3, 35.8) | 1.38 (0.73, 2.62) | 0.326 |
| ujaa | Maxillomandibular | 1052.4  (977.4, 1127.3) | 1026.2  (959.9, 1092.4) | 1.20 (0.60, 2.39) | 0.599 |
| pnsh | Hyoid position | 71.4  (68.1, 74.7) | 73.5  (70.4, 76.6) | 0.74 (0.38, 1.41) | 0.358 |
| c3_msy | Hyoid position | 8.4  (5.8, 10.9) | 11.8  (9.4, 14.2) | 0.55 (0.30, 1.02) | 0.059 |
| c3hm | Hyoid position | 154.0  (146.8, 161.1) | 146.3  (139.6, 153.0) | 1.59 (0.89, 2.85) | 0.121 |
| c3hb | Hyoid position | 39.0  (37.3, 40.7) | 39.2  (37.6, 40.8) | 0.96 (0.48, 1.92) | 0.901 |
| hm | Hyoid position | 41.7  (39.3, 44.0) | 45.9  (43.7, 48.2) | 0.46 (0.25, 0.86) | 0.015^**^ |
| *Models adjusted for age, sex and BMI; ^†^Standardized OR equal to the relative change in odds of being a responder for a 1 SD increase in anatomy measure; **significant (p < 0.05) | | | | | |
